# Novel Colchicine Analogues Target Mitochondrial PT Pores Using Free Tubulins and Induce ROS-Mediated Apoptosis in Cancerous Lymphocytes

**Published:** 2018

**Authors:** Marjan Aghvami, Peyman Eshghi, Mohammad Hadi Zarei, Hadi Arefi, Fatemeh Sattari, Afshin Zarghi, Jalal Pourahmad

**Affiliations:** a *School of Pharmacy, Shahid Beheshti University of Medical Sciences, Tehran, Iran.*; b *Pediatric Congenital Hematologic Disorders Research Center, Shahid Beheshti University of Medical Sciences, Tehran, Iran*.

**Keywords:** B-acute lymphoblastic leukemia, Microtubules inhibitors, Mitochondria, VDAC, Caspase cascade, Apoptosis

## Abstract

B-acute lymphoblastic leukemia (B-ALL) is the frequent pediatric malignity. Chemotherapy is the most practical approaches to deal with such malignancies. Microtubule-targeted agents are one of the most strategic drugs which formerly used in chemotherapy. Although colchicine-binding anti-tubulin agents exhibited promising effects in clinical trials, their exact mechanism of action is not fully understood. In this study, the effects of two newly synthesized of colchicine derivatives were investigated on cell viability of cancerous and normal lymphocytes. The viability test was carried out by MTT assay. Apoptosis vs. necrosis was measured by double staining with annexin V/PI, and caspase-3 as the ultimate mediator of apoptotic measured through the colorimetric assay. Parameters of mitochondrial damage (ROS formation, MMP (Mitochondrial Membrane Potential) decline, mitochondrial swelling, and cytochrome c release following treatment by colchicine derivatives. By focusing on mitochondrial parameters, we showed that following treatment by two newly synthesized colchicine derivatives, apoptosis is triggered in cancerous B-lymphocytes. We demonstrated these compounds could activate apoptosis in cancerous lymphocytes by augmentation of reactive oxygen species (ROS), a decline in mitochondrial membrane potential (MMP), mitochondrial swelling, release of cytochrome c, and also caspase-3 activation. Considering the obtained evidence, these inhibitors could be the new therapeutic strategies in ALL treatment.

## Introduction

B-acute lymphoblastic leukemia (B-ALL) is the most widespread pediatric hematological malignity, further known as the initial childhood cancer-relevant mortality. ALL is a detrimental clonal propagation of lymphoid precursor cells, most generally of the B-cell lineage (B-ALL). ALL is specified by massive production of premature white blood cells, entitled lymphoblast or leukemic blasts. These cells amplitude in bone marrow, prohibit it from making regular blood cells. Besides, they may penetrate into the blood stream and circulate in the whole body; however, due to their immaturity, they are not capable of performing the normal function (1). 

There are several manners to deal with such malignancies, among them chemotherapy is the most beneficial. Microtubule-targeted agents are the most important drugs which are already used in conventional therapy. Microtubules are known as prominent cellular targets in cancer chemotherapy due to their main role in mitosis (2). Microtubules-targeted agents which inhibit microtubule dynamics efficaciously prevent cell cycle progression and finally result in apoptosis. Microtubules inhibitors can be arranged into two major categories: tubulin polymerizers (e.g., taxanes), and depolymerizers (e.g., Vinca alkaloids, colchicines) (3). 

Microtubule inhibitors (MI) are so far used in hematological disorders, such as acute lymphocytic leukemia and Hodgkin lymphoma (4, 5).It has also shown that microtubule inhibitors induce apoptosis in CLL leukemic cells; however, the exact mechanism of cell death is still debatable (6).

Despite their anti-cancer features, many anti-microtubule agents exhibit poor clinical outcomes due to their toxic effects not only on cancerous cells but also on normal cells, resultant several adverse effects (7). On the other side, resistance to chemotherapeutic drugs is always a serious circumstance in the treatment of acute leukemia. Accordingly, it is crucial to investigate new microtubule inhibitors with the specific effect on leukemic cells without the unfavorable impact of normal cells.

Colchicine-binding anti-tubulin agents displayed promising anticancer effects over clinical trials (8).These studies have just concern about clinical consequences, whereas our study emphasis on the connection between mitochondria and microtubules inhibitors to onset the intrinsic pathway of apoptosis. 

Recent researches revealed an interrelation between VDAC the main outer mitochondrial membrane pore and the tubulin (9, 10). Hence, the key role of VDAC in the release of pro-apoptotic molecules from the inter-membrane space to the cytosol is expected.

By consideration of mitochondrial parameters such as generation of reactive oxygen species (ROS), a decline of mitochondrial membrane potential (MMP), mitochondrial swelling, the release of cytochrome c, and also caspase-3 activity, we demonstrated that colchicine derivatives may activate apoptosis through mitochondrial pathways, particularly in human leukemic cells. 

## Experimental

*Chemicals*

RPMI1640 and FBS (Fetal Bovine serum) were purchased from Gibco, Life Technologies, Grand Island, NY. Trypan blue, 2′,7′-dichlorofuorescin diacetate (DCFH-DA), Rhodamine123, bovine serum albumin (BSA), N-(2-hydroxyethyl) piperazine-N′-(2-ethanesulfonic acid) (HEPES), and acridine orange were purchased from Sigma-Aldrich Co. (Taufkirchen, Germany). Ficoll-paque PLUS was obtained from Ge Healthcare Bio-Science Company. The target spiro β-lactams were synthesized in the Department of Pharmaceutical Chemistry, School of Pharmacy, ShahidBeheshti University of Medical Sciences, Tehran, Iran. 

Compound A: 3-(benzo[d] [1,3]dioxol-5-yl)-2-(3,4,5-trimethoxyphenyl)-5-oxa-2-azaspiro[3,4]octan-1-one.

Compound B: 3-(3,4-dimethoxyphenyl)-2-(3,4,5-trimethoxyphenyl)-5-oxa-2-azaspiro[3,4]octan -1-one. 

*Chemistry and Synthesis*

The target spiro β-lactams were synthesized using the Staudinger reaction. Accordingly, the β-lactam ring scaffold was prepared from the appropriately acyl halide and imine precursors. As illustrated in scheme 1, equi-molar amount of imine and triethylamine dissolved in dry toluene under argon gas. Then, the solution was heated to boiling point. To this boiling solution equi-molar amount of tetrahydrofuran-2-carbonyl chloride diluted in toluene was added during 1 h (scheme below). After 24 h refluxing, the reaction mixture cooled and toluene evaporated under vacuum. The residue purified by crystallization in suitable solvent or by flash chromatography.

The structure of synthesized compounds was confirmed by LC-Mass (Liquid chromatography–mass spectrometry), IR spectroscopy (Infrared spectroscopy), and NMR (Nuclear Magnetic Resonance Spectroscopy).

**Figure:**
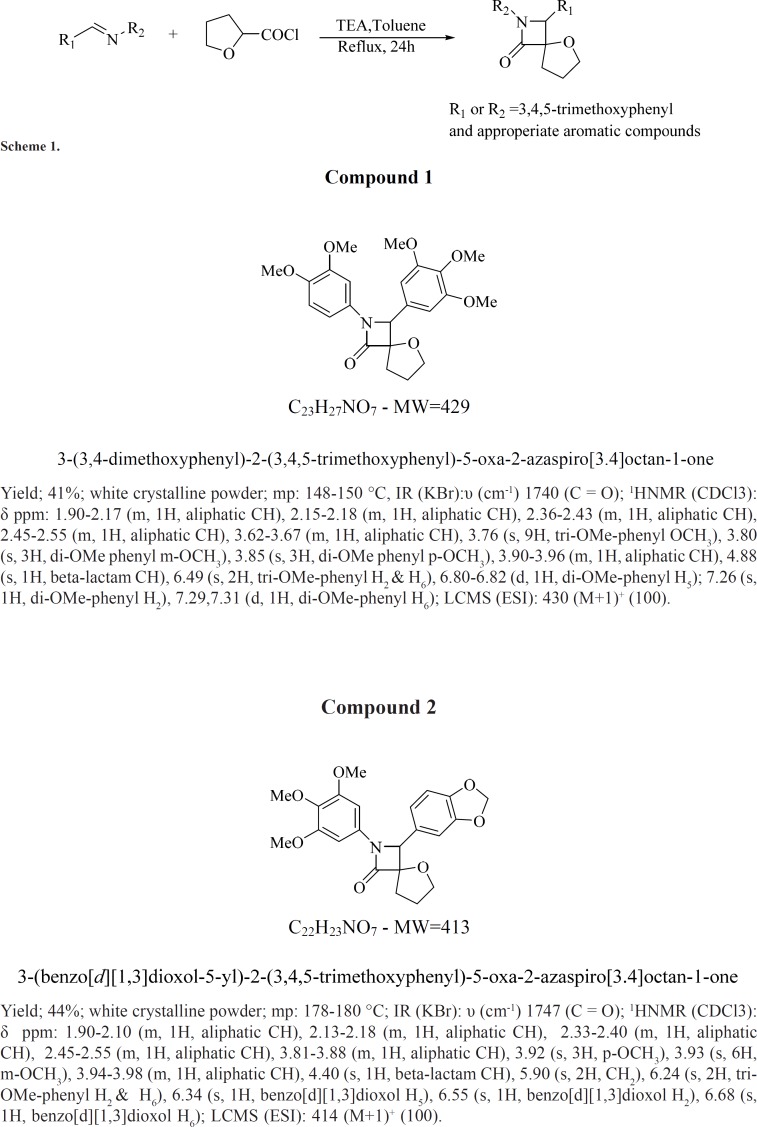


*Selection of healthy donors and patients with ALL*

Twenty ALL patients (10 males and 10 females) aged 2-9 years were enrolled in this investigation. ALL was diagnosed and confirmed according to the definition of the World Health Organization (WHO) classification by specialist. Only those patients without previous treatments within the last six months were comprised in our study. All patients have sampled prior any treatment. Age-matched controls were obtained from 20 healthy donors. This study was approved by the ShahidBeheshti University of Medical Science’s ethics committee, and all the patients and healthy controls signed an informed consent form.

*Cytotoxicity assay*

The effect of colchicine derivatives (A and B) on B-lymphocytes obtained from healthy donors and patients with ALL was investigated using MTT assay (11). Briefly, the cells were seeded in 96-well culture plates at a density of 10^5^ cells per well. The colchicine derivatives were added to various final concentrations in triplicate. The optical density was read at 580 nm wavelength in an ELISA plate reader after 4 h incubation of the plates with MTT in an incubator.

*Determination of cellular energy status*

ATP was measured luminometrically based on luciferin–luciferase bioluminescence reaction (12). Cellular ATP was measured by direct lysis of the cells; in this case, the released ATP reacts with the luciferin-luciferase and produces light with a peak emission at 560 nm. The intensity of light is proportional to the amount of ATP and was measured by Berthold FB12 Luminometer.

*Determination of caspase-3 activity*

Caspase-3 activity was calculated in cell lysate of lymphocytes following different treatments using Sigma’s caspase-3 assay kit (13). This test is dependent upon the hydrolysis of substrate peptide, Ac-DEVD-pNA, through caspase-3. The concentration of the p-nitroaniline (mM) is calculated from the absorbance values at 405 nm.

*Determination of cellular apoptosis*

Apoptosis was determined using *BioVision*Annexin V-FITC Apoptosis Detection Kit. Briefly, cells (5 × 10^5^) were treated with different concentrations of colchicine derivatives. After 12 h the cells were washed with PBS and re-suspended in 500 μL binding buffer. FITC-conjugated annexin V and PI were added according to the manufacturer’s instructions (Biovision, USA). After incubation for 5 min at room temperature, the samples were analyzed on a flow cytometer (Becton–Dickinson) with excitation using a 488 nm argon ion laser.

*Isolation of Mitochondria from B-lymphocytes*

Mitochondria were isolated from the B-ALL lymphocytes by the combination of mechanical lysis and differential centrifugation. Briefly, B-lymphocytes were washed with cold PBS and centrifuged at 450× g. The pellet was re-suspended in cold isolation and the cells were disrupted by homogenization. Non- lysed B-lymphocytes and nuclei were spun down by centrifugation at 1000 ×g for 10 min. The supernatant was further spun at 20000 × g for 25 min. The pellet, designated as the mitochondrial fraction, was suspended in the assay. The isolation of mitochondria was determined by measurement of succinate dehydrogenase (14).

*Succinate Dehydrogenases Activity*

To determine the activity of mitochondrial complex II, succinate dehydrogenases was assayed by measuring the reduction of MTT (15). Briefly, mitochondrial suspensions (1mg protein/mL) were incubated with different concentrations of colchicine derivatives at 37 °C for 1hour. The absorbance at 570 nm was measured with an ELISA reader (Tecan, Rainbow hermo, Austria).

*Determination of ROS formation in Isolated Mitochondria*

The mitochondrial ROS formation was conducted using DCFH-DA. Briefly, isolated mitochondria were placed in respiration buffer and then various concentrations of colchicine derivatives were added. Then, the fluorescence intensity of DCF was measured using fluorescence spectrophotometer at an excitation wavelength of 488 nm and the emission wavelength of 527 nm (16).

*Determination of MMP on Isolated Mitochondria*

Rhodamine 123 dye uptake has been used for mitochondrial membrane potential assessment. The mitochondrial fractions were incubated with 10 μM of rhodamine 123 in MMP assay buffer and then added various concentrations of colchicine derivatives for an hour. The fluorescence was monitored using fluorescence spectrophotometer at the excitation and emission wavelength of 490 nm and 535 nm, respectively (17).

*Determination of Mitochondrial Swelling in Isolated Mitochondria*

Mitochondrial swelling was quantified spectrophotometrically during1 h. This method identifies mitochondrial membrane permeability transition (MMP) with high-amplitude swelling of the mitochondria. Mitochondrial swelling results in a decrease in absorbance monitored at 540 nm (14).

*Determination of Cytochrome c Release from Isolated Mitochondria*

The concentration of cytochrome c was determined through using the Quantikine Human Cytochrome c Immunoassay kit provided by Rand D Systems, Inc. (Minneapolis, Minn). Briefly, a monoclonal antibody specific for human cytochrome c was pre-coated onto the microplate (17).

*Statistical analysis*

Prism 6.01 software was used for all statistical analyses. The results are presented as mean ± SD. Assays were carried out in triplicate. Statistical significance was specified using the one-way and two way (ANOVA) test, followed by the post-hoc Tukey and Bonferroniwhen appropriate. Statistical significance was set at* p *< 0.05. 

## Results

*Cellular Assay*

*Cell viability*

**Figure 1:**
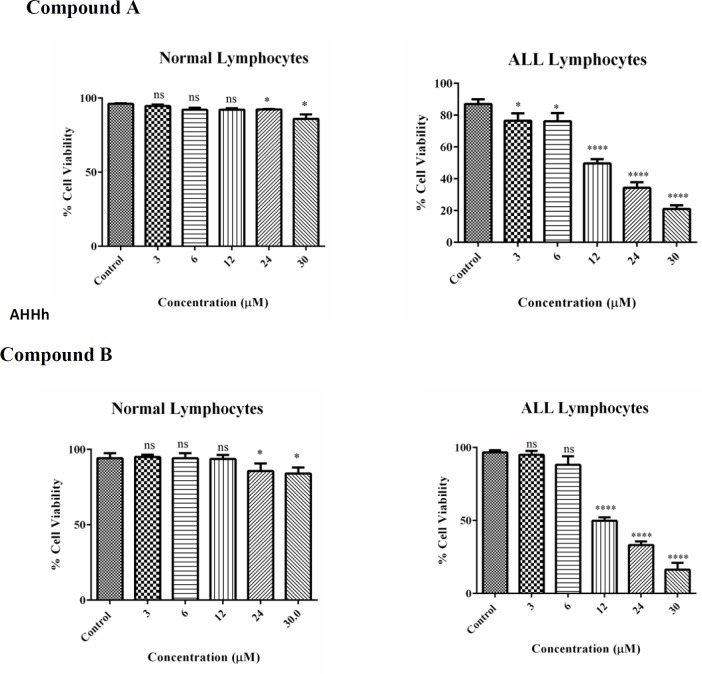
Cell viability, B-lymphocytes from ALL and healthy donors, at 1 × 10^6^ cells/well, were seeded on 96-well plates. Colchicine derivatives (A and B) at 3, 6, 12, 24 and 30 μM concentrations were incubated for 12 h.

As shown in Figure 1 following 12 h a significant reduction in cell viability was noticed for colchicine derivatives (either A or B) at concentrations of 3, 6, 12, 24 and 30 µM only in the B-lymphocytes obtained from ALL patients but not from healthy donors. Our results with MTT assay demonstrated that 12, 24, and 30 µM colchicine derivatives significantly (*p *< 0.001) decreased cell viability.

The absorbance representing the viability of B-lymphocytes was determined by the ELISA reader at 570 nm. The columns represent the viability of B-lymphocytes in both healthy and ALL groups. Data presented as mean ± SD. The significant level was *p* < 0.0001 (n = 6).

*Determination of cellular energy status*

**Figure 2:**
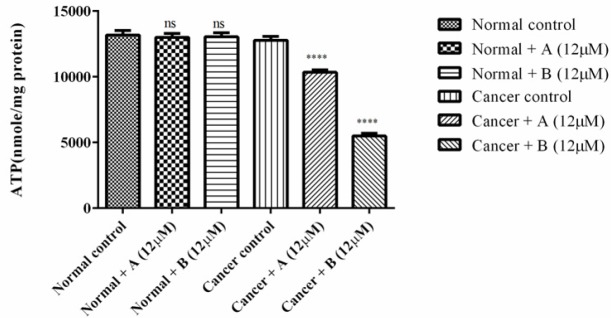
The effect of colchicine derivatives (A and B) on ATP content in both ALL and healthy B-lymphocytes. ATP was measured luminometrically based on luciferin–luciferase bioluminescence reaction. Columns represent ATP (nmol/mg protein) in ALL and healthy B-lymphocytes treated with colchicine derivativesfor 6 h. Values (mean ± S.D.) are from three independent experiments (n = 6) ^****^p < 0.0001

As illustrated in Figure 2 both colchicine derivatives can cause a serious decline in intracellular ATP only in ALL B-lymphocytes but not in normal lymphocytes.

*Determination of Caspase-3 Activity*

**Figure 3:**
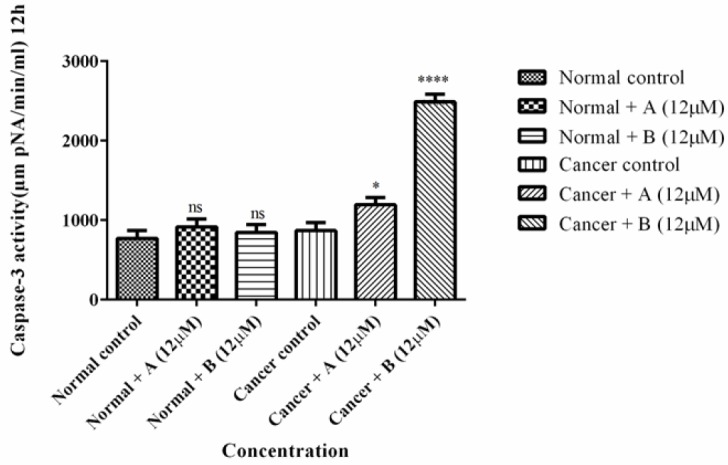
The effect of Colchicine derivatives (A and B) on caspase-3 activity in both ALL and healthy B-lymphocytes. ALL and healthy B-lymphocytes (10^6^ Cells/mL) were incubated in RPMI 1640 medium in conventional condition (37 °C and 5% CO_2_-air) following the addition of Colchicine derivatives (A and B). Caspase-3 activity was determined by Sigma-Aldrich kit. Columns represent caspse-3 activity (µM pNA/min/mL) in ALL and healthy B-lymphocytes treated with colchicine derivativesfor 6 h

As shown in Figure 3 colchicine derivatives significantly (*p *< 0.0001) heightened the activity the essential apoptosis mediator, caspase-3 in ALL B-lymphocytes; however there was not any remarkable impact on healthy B-lymphocytes.

Values (mean ± S.D.) are from three independent experiments (n = 6).^*^p < 0.05, ^****^p < 0.0001.

*Determination of cellular apoptosis*

**Figure 4:**
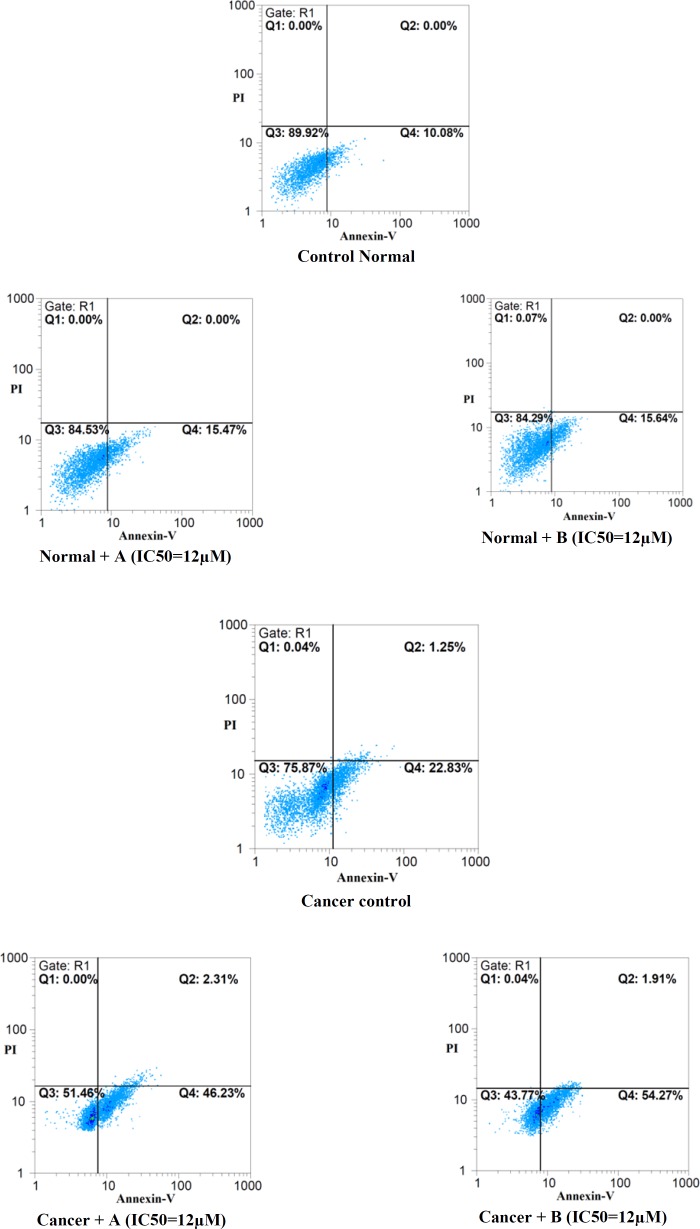
Effects of colchicine derivatives (A and B) on apoptosis in both ALL and healthy lymphocytes.Colchicine derivatives induced apoptosis in ALL but not in normal B-lymphocytes at IC_50_ concentration (3 μM) within 12 h

As demonstrated in Figure 4 staining control group with a combination of FITC-conjugated annexin V and PI showed that great proportion of cells remains intact.

However, both colchicine derivatives in their IC_50_ concentration (12μM) effectively induced apoptosis only in cancerous lymphocytes.

*Mitochondria assay*

*Succinate Dehydrogenase Activity*

**Figure 5:**
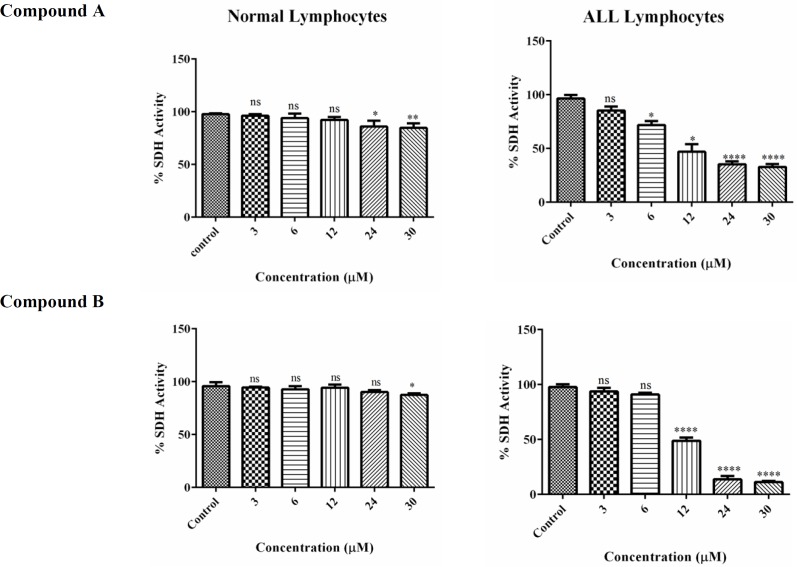
The effect of Colchicine derivatives (A and B) on Succinate dehydrogenase activity in both ALL and healthy B-lymphocytes. The effect of colchicine derivatives (A and B) on succinate dehydrogenase activity in both healthy and ALL mitochondria obtained from human B-lymphocytes were evaluated by MTT assay following 1 h of treatment. Values (mean ± S.D.) are from three independent experiments (n = 6) ^*^p < 0.05, ^**^p < 0.01, ^****^p < 0.0001

In this study, MTT assay was employed to estimate the impact of colchicine derivatives on mitochondrial succinate dehydrogenase activity in mitochondria isolated from lymphocytes of both healthy donors and ALL patients. Colchicine derivatives (6, 12, 24 and 30μM) efficiently inhibited succinate dehydrogenase activity only in ALL mitochondria (Figure 5). The inhibitory effect of these compounds on mitochondrial succinate dehydrogenase in healthy mitochondria was only shown at the highest concentrations of 24 and 30 μM (Figure 5).

*ROS formation assay*

**Figure 6:**
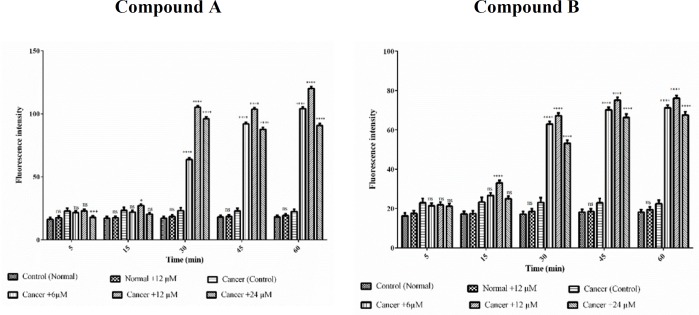
The effect of Colchicine derivatives (A and B) on ROS formation in both healthy and ALL mitochondria. Freshly isolated purified mitochondria were obtained from both healthy and ALL donors incubated with of colchicine derivatives (A and B) for 1 h. Columns represent mean of DCF fluorescence intensity in ALL and healthy B-lymphocytes treated with colchicine derivatives for 0–60 min. Values (mean ± S.D.) are from three independent experiments (n = 6) ^****^p < 0.0001

On account of the fact that ROS have a substantial role in apoptosis, we examined if colchicine derivatives could alter the level of ROS in ALL or healthy mitochondria. As shown in Figure. 6, treatment with colchicine derivatives at 6, 12 and 24 μM for 60 min, significantly influenced ROS generation (*p *< 0.0001) only in ALL mitochondria. Aso,as demonstrated in Figure 6 colchicine derivatives did not induce ROS production in healthy mitochondria at IC_50_ concentration (12μM).

*MMP assay*

**Figure 7:**
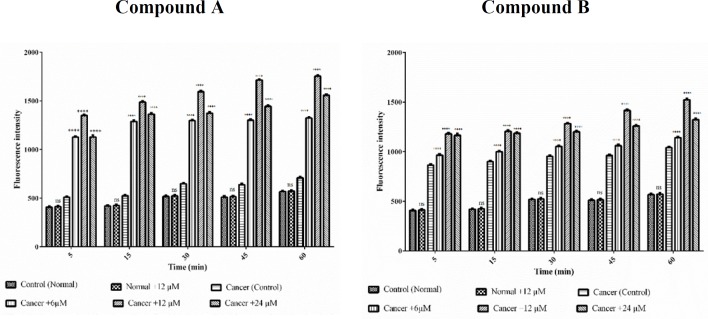
Effects of Colchicine derivatives (A and B) on MMP decline in both ALL and healthy mitochondria. Colchicine derivatives-induced MMP decline in ALL but not in healthy mitochondria. Columns represent mean of Rhodamine123 fluorescence intensity in ALL and healthy mitochondria treated with colchicine derivatives5-60 min. Values (mean ± S.D.) are from three independent experiments (n = 6). ^****^p < 0.0001

We estimate the effects of colchicine derivatives on mitochondrial membrane potential (MMP) as well. Treatment with different concentrations of colchicine derivatives (6, 12 and 24 μM for 60 min) significantly (*p *< 0.0001) decreased MMP only in ALL mitochondria (Figure 7) however this concentration did not alter MMP in healthy mitochondria. 

*Mitochondrial swelling assay*

**Figure 8:**
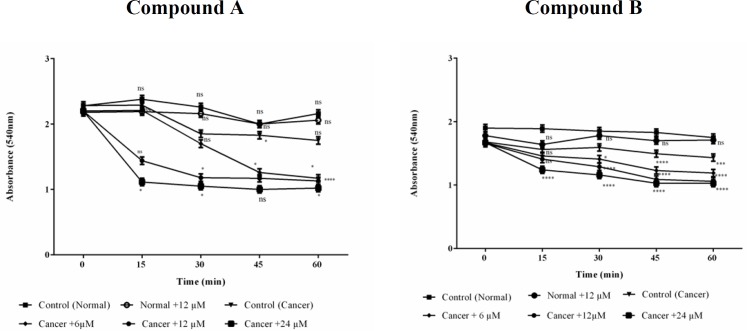
Effects of Colchicine derivatives (A and B) on mitochondrial swelling in both ALL and healthy mitochondria. Addition of colchicine derivatives (A and B) 6, 12 and 24μM induces mitochondrial swelling in ALL but not in healthy B-lymphocyte mitochondria in a concentration depending manner. Mitochondria were suspended in swelling buffer and incubated for 1 h. The mitochondrial swelling was measured by following absorbance (λmax = 540 nm) decrease. Values (mean ± S.D.) are from three independent experiments (n = 6) ^*^p < 0.05,^ **^p < 0.01, ^***^p < 0.001, ^****^p < 0.0001

Mitochondrial swelling was detected by absorbance recording at 540 nm within 60 min of incubation. A tremendous swelling in ALL mitochondria was observed following addition of different concentrations (6, 12 and 24 μM) of colchicine derivatives (Figure 8) without any significant alteration in healthy B-lymphocytes (Figure 8).

*Mitochondrial membrane integrity*

**Figure 9:**
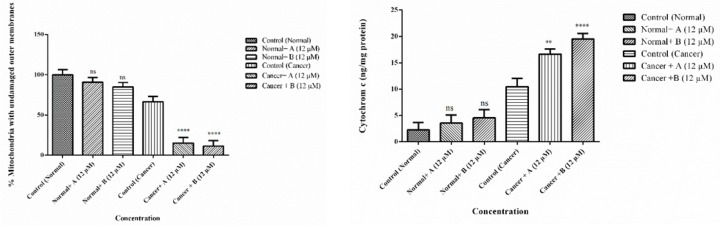
Effects of Colchicine derivatives (A and B) on mitochondrial membrane integrity and cytochrome c release in both ALL and healthy mitochondria. Colchicine derivatives (A and B) (12 μM) cause mitochondrial membrane damage and cytochrome c release in the ALL mitochondria but not in healthy mitochondria. The amount of expelled cytochrome c from mitochondrial fraction into the suspension buffer was determined using human Cytochrome c ELISA kit as described in above. Values (mean ± S.D.) are from three independent experiments (n = 6) ^*^p < 0.05,^ ***^p < 0.001

As shown in Figure 9 colchicine derivatives (A and B) in their IC_50_ concentration (12 μM) caused mitochondrial membrane damage in the ALL mitochondria but not in healthy mitochondria.

*Cytochrome c release*

Since our findings demonstrated that the colchicine derivatives significantly cause mitochondrial membrane damage, mitochondrial swelling and collapse of the MMP which are associated with MPT pores opening. Therefore, release of cytochrome c from mitochondria to cytosol was expected. As demonstrated in Figures 9 colchicine derivatives (12 μM) significantly induced (*p *< 0.001) cytochrome c release only in ALL but not in healthy mitochondria. 

## Discussion

Childhood acute lymphoblastic leukemia (ALL) is a class of malignancy in which bone marrow produces a great number of immature lymphocytes (18). Numerous studies have been performed to overcome the adversity of this malignancy. Despite diver’s recent therapies, pediatric ALL is still considered a disease with a high incidence of relapse and resistance to routine chemotherapy (5). Consequently, it is a serious demand to expand novel agents and new treatment plan for curing ALL. Colchicine site-binding anti-tubulin agents exhibited positive outcomes during clinical trials (8) however, their benefits are limited due to their intense toxic effects.

Although there are several distinguished objectives for the micro tubulin destabilizing drugs, the impact of these drugs on mitochondria is noteworthy (19). Several investigations on both purified organelles and intact cells showed that tubulin is strongly associated with mitochondrial membranes (20). It has been shown that microtubule depolymerization agents, which enhance free tubulin, cause a hyperpolarization of the mitochondrial membrane in tumor cells (21). Besides, another immune precipitation research showed an association between tubulin and VDAC (10, 20). Considering these facts, VDAC (Voltage-Dependent Anion Channel) could be the tubulin-binding site on the mitochondrial outer membrane.

In another study, it was shown that microtubule-targeting drugs effectively hyperpolarize mitochondria and cause the mitochondrial matrix fragmentation and cytochrome c release (19, 22).

Previous studies on cancerous HepG2 cells proved that treatment with colchicine which enhances free dimeric tubulin was accompanied by a loss of mitochondrial inner membrane potential (23). 

Taken together, mitochondria might be the first candidate to explain particular effects of tubulin inhibitors on cancerous cells like ALL B-lymphocytes.

In accordance with previous investigations, we demonstrated that colchicine-like anti-tubulin agents are able to provoke pro-apoptotic mediators′ release only in cancerous B-lymphocytes. Our results revealed that the massive cytochrome c release from cancerous mitochondria in response to colchicine derivatives treatment results in an increase in caspase-3 activity. 

The augmentation of cytosolic cytochrome c subsequent to colchicine derivatives treatment, suggests the key role of mitochondria during the apoptosis procedure.

To realize if the release of cytochrome c from mitochondria is the outcome of membrane depolarization, we treated samples of both cells and isolated mitochondria with colchicine derivatives for 12 h and evaluated their mitochondrial membrane potential in numerous time intervals using fluorescent probe rhodamine 123 (17).

Our investigation with B-ALL lymphocytes and isolated mitochondria also revealed that ROS (Reactive Oxygen Species) intercede cytochrome c release through MPT and detriment of the outer mitochondrial membrane. Our results also demonstrate that tubulin can regulate ATP/ADP flux across mitochondrial outer membrane merely in cancerous B-lymphocytes and not in healthy samples.

In this study, we showed that colchicine derivatives induced cell death through activation of the mitochondrial cell death signaling pathways. To conclude, it is obvious that microtubule inhibitors generally require the caspase cascade to manage the cell death program.

In conclusion, the main findings reported here is that colchicine derivatives act directly on mitochondria isolated from B-ALL lymphocytes but not healthy lymphocytes. Considering that, the high selectivity of colchicine derivatives for leukemic cells in ALL, suggests the use of this inhibitor for designing new therapeutic strategies in ALL treatment. Also, further perception about the mechanisms of action of new microtubule inhibitors has benefits to find the agents with higher efficacy and lower toxicity to overcome cell resistance of common microtubule inhibitors.

**Scheme 2:**
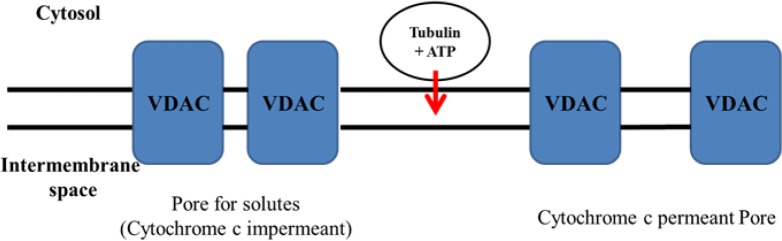
Proposed mechanisms for the selective colchicine derivatives induced mitochondrial targeting in ALL B-lymphocytes
